# The Role of Oral Supplementation for the Management of Age-Related Macular Degeneration: A Narrative Review

**DOI:** 10.3390/jpm14060653

**Published:** 2024-06-18

**Authors:** Angela D’Angelo, Livio Vitiello, Vincenzo Gagliardi, Giulio Salerno, Ilaria De Pascale, Alessia Coppola, Giulia Abbinante, Alfonso Pellegrino, Giuseppe Giannaccare

**Affiliations:** 1 Department of Clinical Sciences and Community Health, University of Milan, 20133 Milan, MI, Italy; angela.dangelo@unimi.it; 2 Eye Unit, “Luigi Curto” Hospital, Azienda Sanitaria Locale Salerno, 84035 Polla, SA, Italy; gmed.vitiellol@aslsalerno.it (L.V.); v.gagliardi@aslsalerno.it (V.G.); gmed.salernog@aslsalerno.it (G.S.); i.depascale@aslsalerno.it (I.D.P.); ccc.coppolaa@aslsalerno.it (A.C.); ccc.abbinanteg@aslsalerno.it (G.A.); al.pellegrino@aslsalerno.it (A.P.); 3 Eye Clinic, Department of Surgical Sciences, University of Cagliari, 09124 Cagliari, CA, Italy

**Keywords:** age-related macular degeneration, AMD, diet, food intake, oral supplementation

## Abstract

The majority of neurodegenerative eye disorders occur with aging and significantly impair quality of life. Age-related macular degeneration (AMD) is the third most common cause of visual impairment and blindness worldwide. One of the most important elements in the pathophysiology of neurodegenerative eye disease is certainly oxidative stress, with neuroinflammation and ocular ischemia which may also be significant factors. Antioxidants, either by food or oral supplementation, may be able to mitigate the deleterious effects of reactive oxygen species that build as a result of oxidative stress, ischemia, and inflammation. Over the past few decades, a number of research works examining the potential adjuvant impact of antioxidants in AMD have been published. In fact, there is not only more and more interest in already known molecules but also in new molecules that can help clinicians in the management of this complex multifactorial disease, such as astaxanthin and melatonin. However, while some studies showed encouraging outcomes, others were conflicting. In addition, more and more attention is also being paid to nutrition, considered a pivotal key point, especially to prevent AMD. For this reason, the purpose of this review is to analyze the main antioxidant molecules currently used as oral supplements for AMD treatment, as well as the role of diet and food intake in this ocular disease, to better understand how all these factors can improve the clinical management of AMD patients.

## 1. Introduction

Age-related macular degeneration (AMD) is the third most common cause of severe irreversible vision loss worldwide and it is considered the leading cause of central blindness in industrialized countries, especially in people over 60 years of age [1]. In fact, prevalence data suggest that AMD affects around 200 million people today and it is expected to rise further to almost 300 million by 2040 [1].

AMD is a multifactorial disorder with multiple genetic and environmental risk factors, including genetic predisposition, older age, smoking, obesity, low dietary intake of several vitamins and minerals (A, C, E, and zinc), low dietary intake of lutein and omega-3 fatty acids, and unhealthy lifestyle related to cardiovascular risk factors [2,3].

In 2001, the Age-Related Eye Disease Study (AREDS) [4] first showed that patients with advanced AMD, or those with at least intermediate AMD (defined as bilateral large drusen with or without pigment changes) [5], could benefit from taking oral antioxidant vitamin and mineral supplements [4].

**Figure 1:**
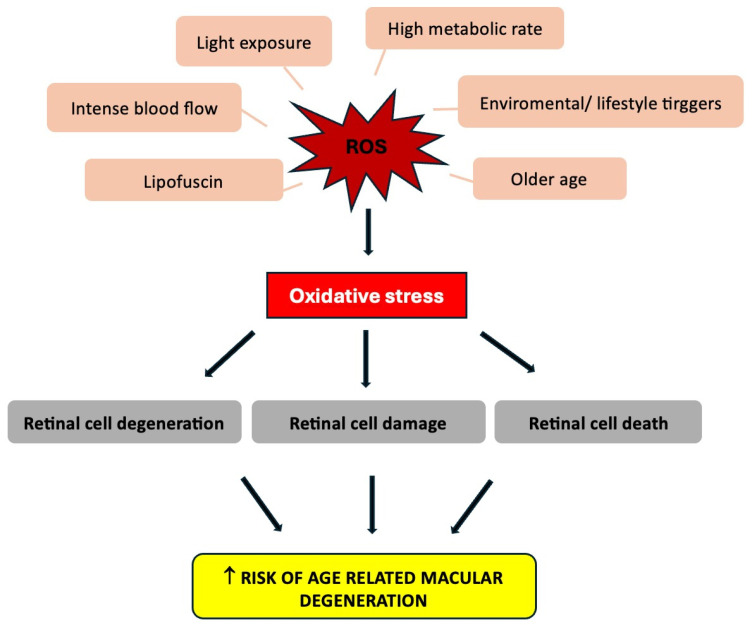
Factors predisposing the formation of reactive oxygen species (ROS), responsible for oxidative stress which can lead to an increased risk of age-related macular degeneration.

Indeed, one pivotal element in the pathophysiology of neurodegenerative eye disease such as AMD is certainly oxidative stress [6] (Figure 1). 

In response to oxidative stress, which can be induced by molecules like lipopolysaccharides, exosomes derived from retinal pigment epithelial cells (RPE) can induce inflammation in the retina and thus contribute to AMD pathogenesis. Among the key genes involved in oxidative stress, interleukin-1 beta (IL-1β), interleukin-6 (IL-6), Nuclear Factor Kappa B (NF-κB), and tumor necrosis factor alpha (TNF-α) are susceptible to upregulation [7], thus triggering an inflammatory cascade that contributes to disease progression [8].

Moreover, neuroinflammation and ocular ischemia also have a significant impact on the disease. Antioxidants, either by food or oral supplementation, may be able to mitigate the deleterious effects of reactive oxygen species (ROS) that build because of oxidative stress, ischemia, and inflammation by reducing the expression of the genes involved in retinal inflammation [6]. 

Over the past few decades, several studies examining the potential impact of antioxidants in AMD have been published [9,10], showing their potential use in AMD management and also as adjuvants to the therapeutic action of intravitreal anti-vascular endothelial growth factor (VEGF) drugs. 

For this reason, the purpose of this narrative review was to explore a wide range of active substances, including not only vitamins and minerals as antioxidants, but also other substances such as saffron, curcumin, and melatonin, which are currently used as oral supplements for AMD. Our work differs from previous studies by including a broader range of these compounds [11]. Additionally, given the growing interest in understanding the impact of dietary factors on AMD progression, we aimed to integrate recent evidence on the role of diet and food intake in the management of this disease. 

## 2. Main Oral Supplements for AMD Management

### 2.1. Lutein and Zeaxanthin

Lutein and zeaxanthin are pigments produced by plants and belong to the carotenoid family of xanthophylls. They are two fat-soluble antioxidants with very similar structures, differing in the location of one double bond in one of the hydroxyl groups. Lutein and zeaxanthin are only biosynthesized by plants, so these substances must be provided to the body through diet. Major sources of these carotenoids include broccoli, kale, spinach, corn, peas, and egg yolks. The recommended daily intake of lutein is about 10.0 mg, while for zeaxanthin, it is 2 mg [12,13,14]. Lutein and zeaxanthin are the only dietary carotenoids that accumulate in the retina, especially in the macula and in the lens of the human eye, and are called macular pigments, responsible for fine detail vision [12,15]. 

Given their accumulation in the retina, researchers have investigated the role of lutein and zeaxanthin in eye health. Their functions include enhancing visual function [16,17], acting as antioxidants to scavenge free radicals and protect the macula from oxidative damage [18,19], and filtering blue light [19,20]. Lutein and zeaxanthin are the most potent antioxidants for the prevention or risk reduction of AMD and various eye-related conditions [21]. Evidence suggests that these xanthophylls may protect the retina from damage and could potentially prevent or slow down the progression of AMD. 

Numerous studies have explored the impact of lutein and zeaxanthin supplementation (both through whole foods and supplements) and their association with AMD. A multicenter case–control study conducted across five ophthalmological centers in the United States was the first epidemiological study to demonstrate a direct relationship between lutein intake and the risk of AMD [22]. Increasing the consumption of foods rich in specific carotenoids, particularly dark green, leafy vegetables, may help reduce the risk of developing advanced or exudative AMD [22]. 

The evidence supporting the protective effects of lutein and zeaxanthin intake against AMD development comes from the Age-Related Eye Disease Study 2 (AREDS2) published in 2013, an important clinical trial conducted in the United States [23]. The original Age-Related Eye Disease Study (AREDS1) was published in 2001 and was one of the first significant clinical trials to assess the association between nutrient intake and the progression of AMD [4]. In this original study, it was demonstrated that a combination of nutrient supplements (500 mg vitamin C, 400 IU vitamin E, 15 mg β-carotene, 80 mg zinc, and 2 mg cupric oxide) taken daily reduces the risk of developing advanced macular degeneration by 25% [4]. However, subsequent findings linking β-carotene to an increased risk of lung cancer among smokers led to revisions in the formulation [24]. Later, AREDS2 included lutein (10 mg) and zeaxanthin (2 mg) along with omega-3 long-chain polyunsaturated fatty acids, eliminating beta carotene and reducing zinc doses in the formulation [23]. While this modified formula did not further reduce the risk of late AMD progression compared to the previous one, the removal of β-carotene and the addition of lutein and zeaxanthin provided similar protective effects without introducing additional risks for smokers [23]. 

These studies have provided valuable clinical guidance on dietary supplementation for AMD patients [4,23]. Evidence also indicates that higher intake of lutein and zeaxanthin may offer significant protection against AMD, especially in individuals with a high genetic risk based on two major AMD genes [25]. A meta-analysis [21] involving seven controlled trials using lutein and zeaxanthin supplements [16,26,27,28,29,30,31,32,33] assessed the impact of this supplementation on visual performance in individuals with established AMD. The results of the meta-analysis suggest that lutein and zeaxanthin supplementation represents a safe and effective strategy for enhancing the visual performance and contrast sensitivity of AMD patients in a dose–response manner [26]. 

Determining effective doses of lutein and zeaxanthin is challenging due to a lack of long-term studies. However, current evidence suggests that higher dietary intake of these compounds may protect against AMD. A diverse diet rich in leafy greens and other foods can help achieve optimal levels of lutein and zeaxanthin, thus supporting eye health. Further research is needed to improve our understanding of lutein and zeaxanthin and establish recommended targets for these eye-protective carotenoids.

### 2.2. Astaxanthin

Astaxanthin (3,3′-dihydroxy-β, β-carotene4,4′-dione), is a carotenoid that belongs to the family of oxygenated derivatives of carotenoids (xanthophylls) present in nature, mainly in marine environments, where it occurs as a red pigment, contributing to the red color of shrimp, lobsters, and shrimp pulp. Astaxanthin is biosynthesized by phytoplankton and microalgae, accumulating in various aquatic species that represent the main dietary sources of this carotenoid. It is a bioactive compound with promising structural and functional characteristics in the prevention of numerous human diseases as well as in maintaining good health [34,35]. 

Unlike most antioxidants, astaxanthin spans across the double layer of the membrane, providing protection against oxidative stress by neutralizing ROS and other free radicals both in the polar (hydrophilic) and nonpolar (hydrophobic) boundary zones of the cell membrane [36]. Astaxanthin has been shown to have antioxidant activity approximately ten times higher than that of zeaxanthin and lutein [37], as well as other carotenoids such as α-carotene, lycopene, and β-carotene [38]. Furthermore, it has been observed that astaxanthin can suppress the activation of NF-κB inflammatory gene expression induced by hydrogen peroxide by inhibiting the intracellular ROS accumulation [39]. Indeed, it has demonstrated multiple beneficial effects, including anti-inflammatory, antioxidant, anti-diabetic, and anti-cancer activities in addition to protective actions for the skin and the nervous and cardiovascular systems [36,37,40]. 

Several recent clinical trials emphasize the potential role of astaxanthin in enhancing eye health, as suggested by the significant improvement observed in the outcomes of various ocular conditions such as age-related macular degeneration, diabetic retinopathy, glaucoma, and cataracts [37].

In a randomized controlled trial, Parisi et al. evaluated the effects of short-term oral supplementation with carotenoids, including astaxanthin, on retinal function in non-advanced AMD patients [41]. Twenty-seven participants were randomly divided into two groups. One group received daily supplementation containing vitamin E (30 mg), vitamin C (180 mg), zinc (22.5 mg), copper (1 mg), lutein (10 mg), zeaxanthin (1 mg), and astaxanthin (4 mg) for 12 months, while the other group received no supplementation. The supplemented group showed improved central retinal function compared to the placebo group, highlighting the potential benefits of carotenoid supplementation in AMD management [41]. In another multicenter, prospective open-label randomized study, 145 patients were randomly divided into two treatment groups [27]. One group received a supplementation containing lutein (10 mg), zeaxanthin (1 mg), astaxanthin (4 mg), and antioxidants/vitamins, while the other group received no dietary supplementation for a duration of two years. Patients treated with lutein/zeaxanthin, astaxanthin, and other nutrients were more inclined to report clinically meaningful stabilization or improvements in visual acuity, contrast sensitivity, and vision-related functions over 24 months compared to untreated subjects [27]. 

Astaxanthin, due to its potent antioxidant activity, can influence choroidal neovascularization (CNV), another factor contributing to AMD. In fact, CNV is associated with oxidative stress and chronic inflammation in ocular tissues, caused by the overexpression of VEGF. Astaxanthin has demonstrated its ability to suppress CNV in murine experimental models, reducing the formation of abnormal new blood vessels in the retina [42]. These positive effects are supported by molecular mechanisms that regulate the production of inflammatory mediators such as ICAM-1, MCP-1, VEGF, IL-6, and VEGF receptors [42]. 

In conclusion, astaxanthin offers promising treatment prospects for combating ocular diseases and supporting eye health. However, to define optimal dosages and formulations, improving bioavailability and obtaining more data from clinical studies are essential, despite its broad safety profile and potential efficacy in various ocular conditions [37,43].

### 2.3. Vitamins and Minerals

High concentrations of certain antioxidant vitamins and minerals in the retina, coupled with the high levels of the carotenoids in the macula, suggest the hypothesis that micronutrient supplementation might protect against AMD. Vitamins A, C, and E are recognized as the most effective for reducing the risk of macular degeneration [4]. Vitamin A is essential for RPE cells of the human retina, while vitamins C and E are known to act as antioxidants. In addition to these vitamins, minerals such as zinc and selenium have also been associated with eye diseases.

AREDS is the first large multicenter randomized, placebo-controlled study initiated by the United States National Eye Institute, designed to investigate the effect of high-dose supplementation with vitamin C and E, beta-carotene, and zinc, either individually or in combination, on the progression of AMD and cataracts [4]. This study enrolled 3640 patients aged between 55 and 80 years diagnosed with AMD. The AREDS1 study showed a statistically significant benefit in consuming the AREDS formula, with a 25% reduced risk of AMD progression over 5 years; although vision loss continued, it occurred at a slower rate with a reduced risk of developing neovascularization [4]. However, the study has several limitations. Firstly, it is unclear to what extent and at what exact dosage vitamin C, vitamin E, and beta-carotene are beneficial for AMD patients because they were administered only in combination and at a single dosage. Additionally, concerns have been raised about the long-term safety of supplementation with dosages that clearly exceed the recommended daily intake [44]. To address some of the original AREDS limitations, the United States National Eye Institute sponsored a second large-scale randomized clinical trial. This study, known as AREDS2, aimed to investigate if additional nutritional supplements beyond AREDS1 supplements could further reduce the risk of progression to advanced AMD [23]. 

Although AREDS1 and AREDS2 are the most comprehensive studies on vitamin supplementation’s impact on AMD progression, smaller-scale studies have also been carried out. However, research findings on the intake of these vitamins and minerals and their association with AMD risk remain conflicting.

#### 2.3.1. β-Carotene and Vitamin A

Beta-carotene is a carotenoid and it is the precursor of vitamin A. Both compounds are liposoluble and excellent antioxidants, capable of counteracting the onset of free radicals [45]. 

In the AREDS1 study, the supplement formulation included beta-carotene at a dose of 15 mg, based on the structural similarities between the components of the macular pigment and beta-carotene [4]. Therefore, beta-carotene was long part of the recommendation for supplementation in AMD patients. However, it is now established that high doses of β-carotene (30 mg per day) increase the risk of lung cancer in smokers [24]. Although the mechanism behind this harmful effect has not been fully elucidated, side effects do not occur in non-smokers or when the carotenoid is administered at lower doses [46]. Based on these data, it was recommended to omit beta-carotene supplementation in smokers with AMD. Therefore, in the formulation of the AREDS2 study, beta-carotene was replaced with lutein/zeaxanthin [23]. 

The protective effect of vitamin A against AMD is demonstrated by epidemiological data from the National Health and Nutrition Examination Survey (NHANES I), which showed that those who consumed higher amounts of fruits and vegetables rich in vitamin A had a reduced risk of any stage of AMD [47]. A prospective population-based cohort study has demonstrated that the sustained consumption of fruits and vegetables containing provitamin A carotenoids can further decrease the risk of AMD [48]. 

However, other studies have not found a significant association between increased dietary vitamin A intake and reduced AMD risk [22,49]. This conflicting evidence highlights the need for additional studies to clarify the relationship between vitamin A and AMD. 

#### 2.3.2. Vitamin C

Vitamin C, chemically known as ascorbic acid, is synthesized by plants and certain mammals. However, humans have lost this ability over evolution making it necessary to obtain vitamin C from dietary sources. Therefore, vitamin C is an essential micronutrient for humans, with the recommended daily intake ranging from 75 mg for adult females to 90 mg for adult males per day [50]. Fruits and vegetables are the primary sources of vitamin C in the diet. Vitamin C plays a crucial role as a powerful antioxidant, contributing positively to redox oxidative pathways, inflammation, maintaining endothelial integrity, and regulating lipoprotein metabolism. 

Vitamin C is plentiful in the retina, including in premature infants, and its potential protective role in age-related retinal diseases is supported by animal studies. Studies in the mid-1980s showed that ascorbic acid, specifically L-ascorbate, can reduce light-induced damage in rat retinas, likely due to its antioxidant properties [51]. Another study that supports the beneficial effect of Vitamin C is conducted by SanGiovanni et al. that found a reduced risk of neovascular AMD in individuals with a high intake of β-carotene, vitamin C, and vitamin E [52]. However, conflicting results were reported by Evans and Lawrenson, who did not find a significant association between vitamin C and the primary prevention of AMD [11]. This was consistent with other studies that also failed to show a clear link between dietary vitamin C intake and reduced AMD risk, as reported by Delcourt et al. [49] and Seddon et al. [22]. The Eye Diseases Control Study noted a significant correlation between an increased intake of green vegetables and a markedly reduced risk of AMD [53]. However, they found that total vitamin C consumption did not show a significant association with reduced AMD risk [22]. Additionally, the study observed that lower plasma levels of vitamin C were linked to a higher risk of AMD, whereas higher plasma concentrations did not offer protection [22]. Furthermore, a 10-year follow-up study did not find significant effects of vitamin C supplementation on AMD risk [54]. 

Overall, while some studies suggest a potential protective role for vitamin C against AMD, the evidence remains inconclusive and further research on a larger scale is necessary to clarify this relationship.

#### 2.3.3. Vitamin E

Vitamin E is a fat-soluble vitamin and comprises four chemical compounds, among which α-tocopherol is considered the most biologically active and an effective scavenger of free radicals [55]. 

The recommended daily dose of 15 mg for adult males is based on α-tocopherol [50]. Photoreceptors and RPE cells, which have a high concentration of vitamin E, respond to increased oxidative stress by increasing their levels of this vitamin [56]. Some evidence supports the idea that vitamin E might offer benefits to those with AMD. Several studies have indicated a correlation between low serum levels of tocopherol and AMD [57,58], suggesting that elevated plasma carotenoid levels may offer protective effects [59]. 

An interventional study evaluated the effect of vitamin E on 1193 subjects, administering 500 IU of vitamin E (equivalent to 335 mg/day of α-tocopherol) or placebo [60]. After 4 years, no significant benefit was found in vitamin E supplementation for AMD prevention or risk reduction [60]. 

Most randomized prospective studies evaluating the effect of vitamin E on AMD have administered vitamin E in combination with other antioxidants, not allowing them to establish the actual role of vitamin E in protecting against AMD. In fact, while high blood levels of vitamin E may be considered protective, evidence regarding the influence of vitamin E alone on the incidence or progression of AMD is currently limited. 

#### 2.3.4. Zinc

Zinc is an essential trace element, and it is the second most abundant transition metal in the human body, following iron [61]. The human body contains approximately 2 g of this element, with high concentrations in bones, skeletal muscles, liver, and eyes. The eye exhibits a relatively high concentration of zinc compared to other organs and tissues, with the peak concentration reaching around 300 µg/g of dry tissue in the RPE [62]. Zinc is not stored in the human body; thus, daily diet is the only way for humans to acquire zinc. It occurs naturally in the form of sulfides or oxides and in food, it is most abundant in meat, saltwater fish, sunflower and pumpkin seeds, bran, wheat, egg yolks, onion, garlic, and tea [63]. The recommended daily intake of zinc for adults ranges from 12 to 15 mg/day [50]. Zinc is generally considered safe, particularly when taken orally, although long-term use, even at low doses, may lead to anemia. There is evidence suggesting that zinc can affect the utilization of other nutrients, particularly copper [64]. 

Zinc acts as a cofactor for hundreds of enzymes across all enzymatic classes and thus participates in a broad range of metabolic functions [65]. Zinc is recognized as important in the pathophysiology of several groups of diseases, including neurodegenerative diseases [62]. Growing evidence suggests that zinc, due to its antioxidant properties, protects cells from the damaging effects of oxidative stress, presumed to be a causative factor in the development of various age-related retinal disorders [66,67].

The first study assessing the effects of oral zinc administration in AMD patients was conducted in 1988 by Newsome et al. [68]. This was a randomized, 2-year, placebo-controlled clinical trial involving 151 subjects with drusen or macular degeneration, finding a statistically significant reduction in visual acuity loss in the group treated with zinc [68]. The study recommended a more definitive trial before making a general recommendation for zinc supplementation in individuals at risk of vision loss from advanced AMD [68]. Contrary to this finding, two epidemiological studies did not confirm this outcome, where zinc supplementation had no short-term effect on the progression of AMD [69] and was not associated with a reduced risk of AMD [70]. After these, the large randomized, placebo-controlled AREDS/AREDS2 studies were published [4,23]. These studies evaluated supplementation with relatively high doses of some vitamins with or without some minerals, including zinc administered as zinc oxide at 80 mg and copper at 2 mg per day. Copper was included in the AREDS formulations along with zinc to prevent copper deficiency anemia, which can occur due to metabolic competition with zinc during supplementation. These studies suggested that zinc supplementation in combination with the AREDS formula and other antioxidants could suppress retinal degeneration [71]. 

In conclusion, zinc supplementation has shown potential benefits for AMD patients in certain studies, with some indicating a slowdown in progression. However, there is no universal consensus on its efficacy, as other studies either found no effect or, in rare cases, even suggested an adverse effect [62].

#### 2.3.5. Selenium

Selenium is an essential trace element in human body, with recommended daily levels set at 55 μg for both adult women and men [50]. Known for its antioxidant properties, recent studies have explored whether selenium can mitigate the risk of AMD, especially through its role in selenium-dependent glutathione peroxidase, which protects cellular lipids from oxidative damage [72]. However, current evidence is inconclusive and does not clearly confirm that selenium can effectively protect the macula from AMD [53]. The role of selenium in protecting against AMD is still unclear due to a lack of case–control studies. Further research is needed to determine if selenium can effectively prevent AMD and associated eye complications.

### 2.4. Omega-3 Fatty Acids

Omega-3 fatty acids are polyunsaturated fatty acids (PUFAs) defined as essential fats because humans lack the enzymatic mechanism necessary to synthesize them [73]. Their primary sources include fatty fish such as mackerel, salmon, trout, and tuna, which are particularly rich in omega-3 eicosapentaenoic acid (EPA) and docosahexaenoic acid (DHA) [74]. Omega-3 PUFA supplements, such as fish oil, are popular for their recognized anti-inflammatory properties [75]. EPA and DHA can modulate the immune response by reducing the production of pro-inflammatory cytokines (TNF-α, IL-1, IL-6, LTB4) and leukocyte activation, and by increasing anti-inflammatory derivatives like PGD2 [76,77]. Their anti-inflammatory mechanisms include altering the composition of phospholipid fatty acids in cell membranes, disrupting lipid rafts, and inhibiting the pro-inflammatory nuclear transcription factor κB activation, thereby reducing inflammatory gene expression and activating anti-inflammatory responses [78]. Although generally safe and well-tolerated, omega-3 fatty acid supplements may exacerbate bleeding and anticoagulation in individuals using anticoagulants [79].

In recent years, there has been growing interest in the potential protective role of omega-3 PUFAs against various retinal diseases linked to inflammation, ischemia, light exposure, oxidative stress, and aging [80]. DHA is a major structural component of the retina, and EPA may play a role as a precursor to signaling molecules with a potential role to influence retinal function [76]. PUFAs have also shown anti-inflammatory and antioxidant properties, anti-angiogenic, anti-vasoproliferative, and neuroprotective effects [76]. 

Studies on animal models of macular degeneration have also demonstrated encouraging outcomes with PUFA supplementation [77,78,79,80,81,82]. Moreover, they reduce pathological angiogenesis across cellular and animal models by influencing numerous angiogenic factors like platelet-derived growth factor (PDGF) and VEGF [80,83]. The AREDS2 trial included, in addition to lutein and zeaxanthin, omega-3 long-chain PUFAs (DHA 350 mg + EPA 650 mg) in the oral formulation for treating the progression to advanced AMD [23]. Overall, there was no additional benefit observed from the addition of these components to the formulation. Although the addition of omega-3 to the AREDS formulation did not prove to be beneficial, it is believed that higher doses of EPA and DHA may have a desirable effect [84]. Additionally, unsaturated fats aid in the absorption of lutein and zeaxanthin [12]. 

In an observational study conducted by Georgiou and Prokopiou, patients with dry AMD were supplemented with EPA and DHA for up to 6 months at doses ranging from 5 to 7.5 g per day, showing a significant improvement in vision, with patients experiencing a gain of at least 15 letters [85].

### 2.5. Curcumin

Curcumin (diferuloylmethane) is the main bioactive component of the popular Indian spice turmeric (*Curcuma longa*) and is a water-insoluble yellowish-orange colored polyphenol. It is isolated from the dried rhizome of *Curcuma longa* L. which belongs to the *Zingiberaceae* family [86,87]. Belonging to the group of phytochemicals, curcumin is considered a bioactive molecule with pleiotropic effects, demonstrating antioxidative, anti-inflammatory, antimicrobial, antimutagenic, and antiproliferative properties [88,89,90]. The mechanism by which curcumin induces its effects is still not fully understood, but it is considered as a nutraceutical substance for the treatment of some chronic diseases such as diabetes, neurodegenerative diseases, pulmonary infectious, rheumatism, and oncological diseases [91,92].

**Figure 2:**
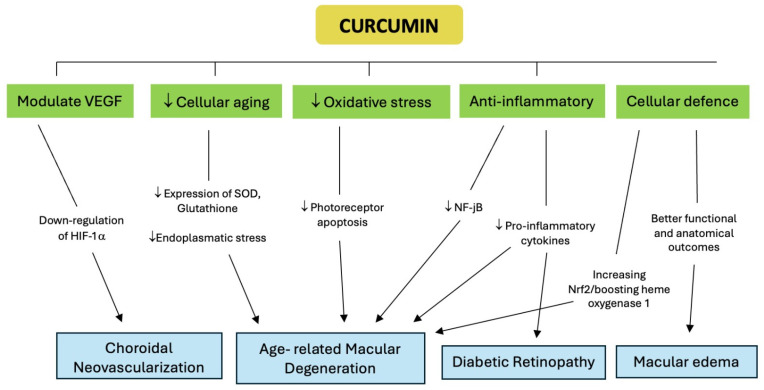
Beneficial mechanisms of action of curcumin on retinal diseases.

Curcumin exhibits promising therapeutic potential in treating various eye conditions, including diabetic retinopathy, chronic anterior uveitis, glaucoma, dry eye syndrome, and AMD [93]. Its mechanisms of action involve reducing apoptosis rates in RPE cells and decreasing overall inflammation by downregulating genes associated with inflammation in AMD [93,94]. Specifically, curcumin’s biological, nutraceutical, and pharmaceutical properties has been linked to its ability to lower levels of TNF-α and proinflammatory interleukins (IL-1, IL-6, IL-8) [86]. Moreover, curcumin protects RPE against oxidative stress and inflammation by activating Nrf2/HO-1 signaling and modulating the ERK pathway, offering potential therapeutic benefits for diabetic retinopathy and AMD [86] (Figure 2). 

These positive effects make curcumin a candidate for treating inflammatory and degenerative retinal eye conditions [86], but research studies on the efficacy of curcumin are mainly limited to in vitro and in vivo studies. 

In an animal study, Mandal et al. observed significant retinal neuroprotection in rats fed diets supplemented with curcumin (0.2% in diet) for 2 weeks [95]. This effect was attributed to curcumin’s ability to downregulate cellular inflammatory genes and inhibit NF-κB activation [95]. Another study found that curcumin reduced the expression of free radicals and gene expression of oxidative biomarkers such as superoxide dismutase (SOD), glutathione, and malondialdehyde [96]. In this study, AMD was modeled by inducing aging in RPE cells with pulsed H_2_O_2_, demonstrating that curcumin led to decreased apoptosis and thus higher cell viability [96]. In addition, a study demonstrated that curcumin has post-transcriptional regulatory effects and can induce gene silencing [97]. Curcumin was also observed to both up-regulate and down-regulate specific microRNAs (miRNAs) that could play a role in regulating the antioxidant system [97]. 

Curcumin, as a PPAR-γ agonist, may help slow AMD progression by reducing microglia’s proinflammatory effects. Saberi et al. found that curcumin activates PPAR-γ, leading to lower production of matrix metalloproteinases (MMPs), particularly MMP-9, which is involved in AMD pathogenesis by promoting RPE cell migration and extracellular matrix degradation [98]. However, exploiting the biomedical potential of curcumin is difficult due to its limited solubility and poor oral bioavailability. The main factors contributing to the low levels of curcumin in plasma and tissues seem to be attributed to its poor absorption, rapid metabolism, and rapid systemic elimination [99]. For this reason, there has been a growing interest on nanoparticles and liposomes to enhance curcumin’s bioavailability [100]. 

In conclusion, curcumin has good tolerance and does not exhibit dose-limiting toxicity and it could be considered safe even at high doses (6 g/day orally for 4–7 weeks) in humans [101]. 

### 2.6. Saffron

Saffron is a spice obtained from *Crocus sativus* L., a plant of the Iridaceae family. Since ancient times, it has been used as an herbal medicine and as a coloring and flavoring spice. Saffron and its major constituents, such as crocetin, crocin, picrocin, and safranal, are natural carotenoids with antioxidant, anti-inflammatory, and neuroprotective effects [102]. These neuroprotective effects have been studied in neurodegenerative diseases such as Alzheimer’s and Parkinson’s [103] and in ocular diseases including AMD, glaucoma, and other retinal diseases. An increasing number of studies have explored the effects and mechanistic pathways of saffron and its compounds to assess their potential therapeutic use in ocular diseases. Most studies showed that saffron has a strong antioxidant activity, due to its carotenoids, especially crocin, protecting biomolecules from free radicals [104]. Additionally, saffron components exhibited anti-inflammatory and antiapoptotic effects possibly by inhibiting apoptosis mediated by caspase after retinal damage [105,106]. Moreover, crocin and crocetin are also known to enhance oxygen diffusion and improve ocular blood flow in the retina and choroid, which are crucial factors in AMD progression [107]. Considering AMD patients, six clinical studies assessed vision-related parameters after oral saffron supplementation [108,109,110,111,112,113]. All these studies showed significant improvement in visual acuity across different saffron dosages (ranging from 20 to 50 mg daily), even with short-term supplementation of around three months [108,109,110,111,112,113].

It is not possible to make a direct quantitative comparison because the above-mentioned studies had differences in formulation, dosage, intervention length, test methods, and outcome measures.

Longer-term data are available from only two non-randomized control trials, both showing improvements within three months of saffron supplementation (20 mg daily), with a stabilization over time [109,110].

### 2.7. Melatonin

Melatonin (N-acetyl-5-methoxytryptamine) is a neurohormone produced by the pineal gland in the human brain. Is a ubiquitous biomolecule in nature, also produced by other species of animals, plants, and microorganisms [114]. Additionally, this hormone is synthesized in human ocular tissues, including the retina and ciliary body, which have specific melatonin receptors [115]. Both melatonin and melanopsin, a regulator of melatonin synthesis in the eyes, are produced in the human lens [116]. Melatonin controls the circadian rhythm and sleep–wake regulation. Furthermore, it regulates various physiological functions including immune response, retinal physiology, sexual behavior, body temperature, and aging processes [117]. Melatonin acts as a scavenger for free radicals, playing a crucial role in detoxifying ROS. This protective action not only shields cells from damage caused by oxidative stress but also regulates the transcription of antioxidant genes through Nrf2 activation [118,119]. Additionally, melatonin influences several other molecular pathways including inflammation, apoptosis, angiogenesis, and autophagy [120]. Recent studies suggest that melatonin may decrease inflammation, oxidative stress, and apoptosis in the retina and increase viability of RPE cells as well as of photoreceptors, indicating its potential role in preventing and treating AMD [121,122]. One study reported that AMD patients have lower levels of nocturnal melatonin production compared to age-matched controls [123]. This could suggest that the decreased synthesis of melatonin may contribute to oxidative stress and the onset of AMD. Moreover, another study showed that the daily use of 3 mg of exogenous melatonin, for 3 months, in one hundred patients with AMD could protect the retina and delay AMD onset [124].

Phagocytic and autophagic processes in RPE cells are influenced by circadian rhythms [125], but the specific role of melatonin is not yet clear. Some studies indicated that melatonin may regulate photoreceptor phagocytosis [126], while others did not confirm this relationship. Retinal circadian clocks protect photoreceptors by reducing melatonin secretion during light exposure [127].

Melatonin also protects RPE cells from light-induced apoptosis through the aggregation of melanosomes and the absorption of light by melanin, which acts as an antioxidant [128]. Melatonin plays a crucial role in protecting the blood-retinal barrier in AMD-associated neovascularization, triggered by the accumulation of ROS [129]. This molecule may reduce the levels of VEGF and nitric oxide in the retina [129], thus preventing degradation of the blood–retinal barrier. Such action of melatonin suggests its potential effect in the treatment of AMD and suggests that its administration, especially in combination with antiangiogenic agents, could increase the efficacy of AMD therapy [119].

## 3. Role of Diet and Food Intake

In the last few years, in addition to all the aforesaid molecules (Table 1), there has been a high interest in the role of diet and food intake since they are considered as modifiable factors for preventing or slowing the progression of AMD [130,131].

**Figure 3:**
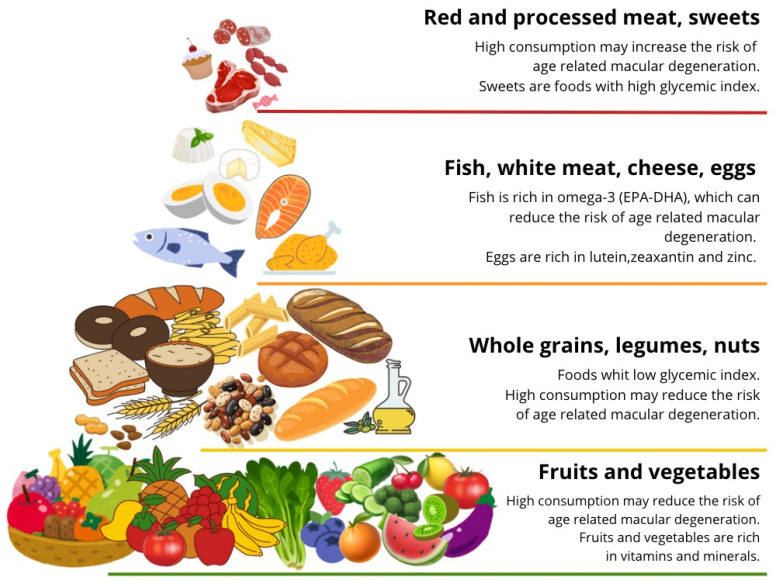
Simplified schematic representation of the food pyramid showing the possible role of food intake on age-related macular degeneration.

Recent studies have highlighted the relationship between the Mediterranean diet and AMD [132,133,134]. The Mediterranean diet is characterized by a high intake of vegetables, fruits, legumes, grains, and nuts; moderate consumption of poultry, dairy and fish; limited consumption of red meat; and low to moderate amounts of red wine may be consumed. Olive oil is used instead of butter as the main condiment fat. High adherence to this dietary pattern has been associated with a lower risk of developing AMD [133,134] and has also been shown to be beneficial for reducing the progression of late AMD [135]. This dietary approach could potentially mitigate oxidative stress and inflammation and provide a shield against AMD progression. The consumption of specific foods also appears to protect against the risk of AMD, such as vegetables rich in carotenoids, especially dark green leafy vegetables [22,135], and fatty fish containing omega-3 fatty acids [82,136]. Conversely, high consumption of foods like animal fats containing omega-6 fatty acids [137] and red and processed meat [138] should be minimized to reduce the risk of AMD progression (Figure 3). 

Table 2 provides a more in-depth overview of the potential role of diet and food intake in AMD. 

Encouraging dietary improvements could help reduce AMD risk by focusing on nutrient-rich foods and avoiding those that cause oxidative damage. Providing nutritional guidance alongside standard treatments could be important for managing AMD effectively.

## 4. Discussion

This review focused on the preventive qualities and effects of several oral supplements as well as food intake for managing AMD. By shielding RPE cells from oxidative stress and preventing them from apoptosis, most of the oral supplements examined in this review provide therapeutic benefits through primarily antioxidant actions. While these supplements cannot replace traditional medical and surgical treatment, they can have a positive synergistic effect that boosts the efficacy of gold-standard therapies and aids in stabilizing the patient’s general state of health.

In fact, the growing use of these oral supplements in clinical practice for the management of AMD has aroused considerable interest on the part of researchers to better understand their potential beneficial effects for the treatment of this ocular disease, adding them to conventional therapy characterized by intravitreal anti-VEGF drugs.

Considering oral supplements, all have shown substantially protective activities towards the retina in case of AMD, both in reducing the risk of development and progression of the disease. Furthermore, except for curcumin, all of these substances have moderate to high bioavailability when taken orally, with a good safety and tolerability profile. In fact, excluding curcumin and zinc, which showed a greater interaction with other drugs and a greater onset of undangerous gastrointestinal disorders, all molecules have demonstrated minimal toxicity in the clinical studies published in the literature.

While lutein and zeaxanthin are well recognized as retinal protective molecules, having already been included in the AREDS2 study [23], of particular interest are certainly astaxanthin, EPA, and DHA, which have shown very promising effects thanks to their anti-inflammatory, anti-angiogenic, anti-vasoproliferative and neuroprotective properties. In particular, all these molecules can exert their beneficial effects by modulating the immune response, thus reducing the production of pro-inflammatory cytokines and leukocyte activation, together with the increase of anti-inflammatory derivatives [76,77].

In addition to oral supplements, diet and food intake have also recently attracted increasing interest in the ophthalmology field, in particular in the clinical management of degenerative diseases such as AMD. In fact, several studies in the literature have demonstrated the protective effects of some foods, such as fish, fruit, and vegetables, and the harmful effects of others, such as red and processed meat, alcohol, and all foods with a high glycemic index. However, more scientific evidence is needed to better establish the relationship between AMD and diet, and to better understand the potential benefits of healthy food for the management and the prevention of this ocular disease.

The narrative and non-systematic nature of this review, together with the fact that it was composed using only one scientific database (PubMed), could be considered limitations of this study. Furthermore, only some of the main oral supplements that are currently used in clinical practice to control AMD have been reviewed in this study.

## 5. Conclusions

The integrative use of antioxidants, vitamins, organic compounds, and micronutrients, along with a balanced and healthy diet, may be beneficial for the treatment of AMD, as well as glaucoma and other ocular disorders [143,144,145].

However, effective oral supplementation strategies are challenging to design due to a number of limiting factors, including the complexity of the anatomy and tissues of the visual system, the timing of patient enrollment in clinical trials, the lack of valid endpoints, and our incomplete understanding of the underlying molecular causes of neurodegenerative diseases, including AMD.

For this reason, more investigations and even large-scale multicenter clinical trials are continuously required to confirm the efficacy of these oral supplements and to gain a deeper understanding of their mechanisms of action to fully utilize their therapeutical properties as adjuvant therapeutic alternatives in treatment regimens for AMD, in addition to the promotion of a healthy diet, given the potential benefits it can bring to AMD patients.

## Figures and Tables

**Table 1:** Overview of the main effects of the discussed oral supplement compounds for the management of age-related macular degeneration.

Active Compounds	Daily Therapeutic Dosage	Prescription Duration	Effects	Bioavailability	Adverse Effect and Contraindications	References	Clinical Trial Number
Lutein	1–20 mg	Weeks–months	Antioxidant activity Improvement of visual function	Moderate	No adverse effects demonstrated	[23,31,32,33]	NCT00345176 NCT00763659 NCT01042860 ISRCTN94557601
Zeaxanthin	2–10 mg	Weeks–months	Antioxidant activity Improvement of visual function	Moderate	May give the skin a golden-yellow color in people with fair complexions	[10,23,31,33]	NCT01527435 NCT00345176 NCT00763659 ISRCTN94557601
Astaxanthin	4–20 mg	Months	Antioxidant activity Anti-inflammatory effects	Low/Moderate	Minimal toxicity	[37,42]	
Vitamin A	700–900 mcg	Weeks	Antioxidant activity	Moderate/High	Hypervitaminosis, with possible permanent damage to the liver and spleen	[4]	NCT00000145
Vitamin C	100–500 mg	Months	Antioxidant activity	High	Digestive problems, vomiting, diarrhea, gastritis; may increase the risk of kidney stones in men	[23,33]	NCT00345176 ISRCTN94557601
Vitamin E	400–500 UI	Months	Antioxidant activity	High	Digestive problems, vomiting, nausea; may increase blood pression and reduce thyroid hormones	[23,33]	NCT00345176 ISRCTN94557601
Zinc	2–80 mg	Weeks–months	Antioxidant activity	High	Vomiting, nausea or diarrhea, irritability, drowsiness, anemia, dizziness; may cause copper deficiency anemia	[23,33]	NCT00345176 ISRCTN94557601
Selenium	50–200 μg	Weeks–months	Antioxidant activity	High	Digestive problems, vomiting, diarrhea, nausea	[50,72]	
Eicosapentaenoic acid Docosahexaenoic acid	350 mg–7.5 g	Weeks–months	Anti-inflammatory effects Antioxidant properties Anti-angiogenic and anti-vasoproliferative effects Neuroprotective effects	Moderate	May increase the risk of bleeding	[23,31]	NCT00345176 NCT00763659
Curcumin	500 mg–3 g	3–4 weeks	Possible prevention of retinal pigment epithelium cell death Anti-inflammatory effects Antioxidant activity	Very Low	Minimal toxicity, predominantly gastrointestinal upsets; may interfere with antibiotics, cardiovascular drugs, anticancer drugs, and antidepressants	[86]	
Saffron	20–30 mg	Weeks–months	Anti-inflammatory effects Antioxidant activity	Moderate	Mood disorders, lethal dose of approximately 20 g; May interfere with cardiovascular drugs	[9,111]	ACTRN12612000729820 IRCT201205219820N1
Melatonin	3–20 mg	Weeks–months	Antioxidant activity Anti-inflammatory effect Possible increase in RPE cell viability	Moderate	Drowsiness; may enhance sedative effects of some drugs such as benzodiazepines	[119,124]	

**Table 2:** Overview of the possible role of diet and food intake in age-related macular degeneration.

Dietary Factor	Possible Effects on AMD	General Considerations	References
Mediterranean diet	Lower risk of developing AMD Lower risk of AMD progression	High consumption of fruits, vegetables, legumes, whole grains and nuts; moderate consumption of fish, poultry and dairy; limited consumption of red meat; olive oil used as main fat; low to moderate amounts of red wine	[131,132,133,134]
Western diet	Increased AMD prevalence	Higher intake of red meat, processed meat, high-fat dairy products, fried potatoes, refined grains	[135]
High glycemic index diets	Increased risk of early AMD	Glycemic index is a measure that ranks carbohydrate-containing foods based on their impact on blood sugar levels over a 2 h period. Pure glucose is assigned the reference GI of 100. High consumption of foods with high GI (>70): white bread, potatoes, white rice, cereals, honey, and refined sugar Low consumption of foods with low GI (<55): whole fruit and vegetables, whole wheat bread, pasta, oats, bran, legumes, milk, and yoghurt	[139,140]
Vegetables and fruits	Lower risk of AMD	200 g per day of vegetables, in particular dark green leafy vegetables (broccoli, spinach…) and fruit two times per day	[22,48]
Fish and omega-3	Reduced risk of AMD	One or more servings of fish per week	[48,136,137]
Red and processed meat	Increased risk of developing AMD	High consumption especially of processed meat (salami or continental sausages)	[138]
Alcohol	Higher risk of development AMD	More than two drinks a day	[141,142]

AMD: Age-related macular degeneration; GI: glycemic index.

## Data Availability

The data can be shared upon request.
